# Associations of Cigarette Smoking and Polymorphisms in Brain-Derived Neurotrophic Factor and Catechol-*O*-Methyltransferase with Neurocognition in Alcohol Dependent Individuals during Early Abstinence

**DOI:** 10.3389/fphar.2012.00178

**Published:** 2012-10-11

**Authors:** Timothy C. Durazzo, Kent E. Hutchison, Susanna L. Fryer, Anderson Mon, Dieter J. Meyerhoff

**Affiliations:** ^1^Center for Imaging of Neurodegenerative Diseases, San Francisco VA Medical CenterSan Francisco, CA, USA; ^2^Department of Radiology and Biomedical Imaging, University of CaliforniaSan Francisco, CA, USA; ^3^University of ColoradoBoulder, CO, USA

**Keywords:** cigarette smoking, brain-derived neurotrophic factor, catechol-*O*-methyltransferase, neurocognition, alcohol dependence

## Abstract

Chronic cigarette smoking and polymorphisms in brain-derived neurotrophic factor (BDNF) and catechol-*O*-methyltransferase (COMT) are associated with neurocognition in normal controls and those with various neuropsychiatric conditions. The influence of BDNF and COMT on neurocognition in alcohol dependence is unclear. The primary goal of this report was to investigate the associations of single nucleotide polymorphisms (SNPs) in BDNF Val66Met (rs6265) and COMT Val158Met (rs4680) with neurocognition in a treatment-seeking alcohol dependent cohort and determine if neurocognitive differences between non-smokers and smokers previously observed in this cohort persist when controlled for these functional SNPs. Genotyping was conducted on 70 primarily male treatment-seeking alcohol dependent participants (ALC) who completed a comprehensive neuropsychological battery after 33 ± 9 days of monitored abstinence. After controlling for COMT and BDNF genotypes, smoking ALC performed significantly worse than non-smoking ALC on the domains of auditory-verbal and visuospatial learning and memory, cognitive efficiency, general intelligence, processing speed, and global neurocognition. In smoking ALC, greater number of years of smoking over lifetime was related to poorer performance on multiple domains after controlling for genotypes and alcohol consumption. In addition, COMT Met homozygotes were superior to Val homozygotes on measures of executive skills and showed trends for higher general intelligence and visuospatial skills, while COMT Val/Met heterozygotes showed significantly better general intelligence than Val homozygotes. COMT Val homozygotes performed better than heterozygotes on auditory-verbal memory. BDNF genotype was not related to any neurocognitive domain. The findings are consistent with studies in normal controls and neuropsychiatric cohorts that reported COMT Met carriers demonstrated better performance on measures of executive skills and general intelligence. Results also indicated that the poorer performance of smoking compared to non-smoking ALC across multiple neurocognitive domains was not mediated by COMT or BDNF genotype. Overall, the findings lend support to the expanding clinical movement to make smoking cessation programs available to smokers at the inception of treatment for alcohol/substance use disorders.

## Introduction

A number of premorbid and/or comorbid factors may contribute to the pattern and magnitude of neurocognitive abnormalities demonstrated by those with alcohol use disorders (AUD; Parsons and Nixon, 1993; Oscar-Berman, 2000; Sher et al., 2005; Rourke and Loberg, 2009). In our previous work assessing the neurocognitive consequences of AUD, we investigated the influence of chronic cigarette smoking, sociodemographic factors, alcohol consumption levels, as well as comorbid substance abuse, psychiatric and medical conditions (Durazzo et al., 2006, 2007b,c, 2008, 2010a). Among these variables, chronic cigarette smoking was the sole factor that consistently and robustly predicted neurocognition in our AUD participants. Specifically, chronic smoking was associated with significantly poorer performance on measures of executive skills, processing speed, and learning and memory. Additionally, longer duration of smoking over lifetime in these studies was consistently related to poorer performance on multiple domains of neurocognition after controlling for age, alcohol consumption, and other potentially mediating variables.

Human neurocognition is a complex phenotype that is a function of psychosocial, environmental, biological, and genetic factors. With respect to genetic factors, multiple studies have reported that the Val66Met single nucleotide polymorphism (SNP) of the brain-derived neurotrophic factor (BDNF; rs6265) and the Val158Met SNP of the catechol-*O*-methyltransferase (COMT; rs4680) genes are associated with several domains of neurocognitive functioning. Specifically, studies have reported that the BDNF Met allele carriers (i.e., Val/Met, Met/Met) of the BDNF demonstrated poorer verbal memory (Egan et al., 2003; Hariri et al., 2003; Dempster et al., 2005; Tan et al., 2005; Schofield et al., 2009), processing speed (Miyajima et al., 2008; Raz et al., 2009), and general intelligence (Tsai et al., 2004; Miyajima et al., 2008) in controls and individuals with various neuropsychiatric conditions (e.g., schizophrenia). The observed relationships between BDNF genotypes and neurocognition, however, were not uniform across all studies (Harris et al., 2006; Savitz et al., 2006). For COMT, studies with controls and individuals with various neuropsychiatric conditions reported that Met homozygosity was related to better performance on measures of executive skills, working memory, and general intellectual functioning. Alternately, several studies found no relationship between COMT genotype and neurocognition and some reported Val homozygosity was associated with better neurocognitive performance (for review see Savitz et al., 2006; Barnett et al., 2008; Dickinson and Elvevag, 2009; Enoch et al., 2009; Wishart et al., 2011). While the cumulative body of research appears to suggest COMT Met homozygosity is generally associated with better performance on working memory and executive function tasks, the influence of the COMT Val158Met polymorphism on neurocognition has yet to be fully elucidated (Barnett et al., 2008; Goldman et al., 2009). Overall, the majority of research on BDNF has focused on memory function and for COMT on measures of executive skills and working memory in healthy controls and individuals with neuropsychiatric disorders (e.g., schizophrenia-spectrum and bipolar disorders). We are not aware of any study that specifically investigated the association of BDNF and COMT polymorphisms with neurocognition in AUD. Therefore, it is unclear to what extent polymorphisms in BDNF and COMT are related to neurocognitive function in AUD.

The primary goal of this report was to investigate the associations of SNPs in BDNF Val66Met (rs6265), COMT Val158Met (rs4680) with neurocognition in our treatment-seeking alcohol dependent participants and determine if neurocognitive differences between non-smokers and smokers previously observed in this cohort persist when controlled for these functional SNPs. We predicted that smoking alcohol dependent participants compared to non-smokers perform significantly worse on the domains of executive skills, processing speed, and learning and memory after controlling for BDNF and COMT genotypes, alcohol consumption, age, and predicted premorbid intelligence. We also hypothesized that the inverse relationships between lifetime years of smoking and neurocognitive performance we observed in our previous studies are independent of the effects BDNF and COMT polymorphisms in the current study cohort. Finally, we predicted that BDNF Val homozygotes perform significantly better than Val/Met heterozygotes and COMT Met homozygotes show better performance than Val homozygotes on measures of executive skills, learning, memory, and processing speed, after controlling for smoking status, alcohol consumption, age, and predicted premorbid intelligence.

## Materials and Methods

### Participants

Individuals seeking treatment for AUD (*n* = 70; four females) were recruited from the VA Medical Center Substance Abuse Day Hospital and the Kaiser Permanente Chemical Dependence Recovery Program outpatient clinics in San Francisco. All participants provided written informed consent prior to study according to the Declaration of Helsinki, and the informed consent document and procedures were approved by the University of California San Francisco and the San Francisco VA Medical Center. Participants were between the ages of 28 and 68 at the time of study and all met DSM-IV criteria for alcohol dependence (95% with physiological dependence). The alcohol dependent participants (ALC) completed a comprehensive neuropsychological assessment battery after 33 ± 9 days of monitored abstinence. Smoking (*n* = 39) and non-smoking (*n* = 31) ALC did not differ in the duration of abstinence prior to assessment. All smoking ALC were actively smoking at the time of assessment and no participant changed their cigarette consumption from the onset of abstinence to the time of assessment. Five non-smoking ALC reported a previous history of chronic smoking, with four quitting more than 8 years and one more than 3 years prior to enrollment. The performance of the former smokers was within ±0.5 standard deviations of the non-smoking ALC group mean across neurocognitive domains. The vast majority of ALC in this study were participants in our previous research (Durazzo et al., 2008, 2010a). Demographics, indices of alcohol consumption, smoking severity, depressive and anxiety symptomatology, and frequency of medical, psychiatric, and substance use comorbidities for ALC are given in Table 1.

**Table 1:** **Participant demographics and clinical measures**.

Measure	ALC (*n* = 70)
Age	51.0 ± 10.0
Education	14.0 ± 2.2
Days abstinent	33 ± 9
AMNART	114 ± 9
1-year average drinks/month	398 ± 206
8-year average drinks/month	314 ± 163
Lifetime average drinks/month	208 ± 100
Months of heavy drinking	259 ± 116
Age onset heavy drinking	26 ± 10
FTND	5.5 ± 2.7
Cigarette pack years	25 ± 18
Lifetime years of smoking	25 ± 12
Beck Depression Inventory	11.1 ± 9.0
STAI-trait	43.1 ± 11.0
% smokers	56
% with psychiatric comorbidity	44
% with substance comorbidity	24
% with medical comorbidity	44
GGT	44 ± 25
Prealbumin	27 ± 6

*AMNART, American National Adult Reading Test; FTND, Fagerstrom Test for Nicotine Dependence; GGT, gamma glutamyltransferase, normal range 7–64; institutional units; prealbumin (proxy measure of nutritional status), normal range 18–45 mg/dl; STAI, State-Trait Anxiety Inventory; (mean ± SD)*.

Primary inclusion criteria were current DSM-IV diagnosis of alcohol dependence or abuse, fluency in English, consumption of greater than 150 alcoholic drinks per month (one alcoholic drink equivalent = 13.6 g pure ethanol) for at least 8 years prior to enrollment for men, and consumption of greater than 80 drinks per month for at least 8 years prior to enrollment for women. Primary exclusion criteria are fully detailed in our previous work (Durazzo et al., 2004). In summary, no participant had a history of a neurologic (e.g., non-alcohol-related seizure disorder, neurodegenerative disorder, demyelinating disorder; traumatic brain injury with loss of consciousness >15 min), general medical (e.g., myocardial infarction, Type-1 diabetes, cerebrovascular accident), or psychiatric (e.g., schizophrenia-spectrum, bipolar disorder, post-traumatic stress disorder, substance dependence within 5 years prior to study) conditions known or suspected to influence neurocognition. The following comorbidities were permitted due to their high prevalence in AUD (Gilman and Abraham, 2001; Stinson et al., 2005): hepatitis C, type-2 diabetes, hypertension, unipolar mood (major depression, substance-induced mood disorder), and anxiety (generalized anxiety disorder, panic disorder). ALC who met DSM-IV criteria for current or past substance abuse were included. Current opioid replacement therapy (e.g., methadone) was exclusionary.

### Medical, psychiatric, substance, and drinking history assessment

Participant medical history was obtained from self-report and confirmed via available medical records. Participants completed the Structured Clinical Interview for DSM-IV Axis I disorders, Patient Edition, Version 2.0 (SCID-I/P; First et al., 1998), and standardized questionnaires assessing lifetime alcohol consumption (Lifetime Drinking History, LDH; Skinner and Sheu, 1982; Sobell et al., 1988) and substance use (in-house questionnaire assessing substance type, and quantity and frequency of use). From the LDH we derived average number of alcohol-containing drinks per month over 1 and 8 years prior to enrollment, average number of drinks per month over lifetime, number of lifetime years of regular drinking (i.e., consuming at least one alcoholic drink per month), number of months of heavy drinking (i.e., total number of months over lifetime of drinking in excess of 100 drinks per month), age of onset of heavy drinking and total kilograms of ethanol consumed over lifetime. Participants completed self-report measures of depressive (Beck Depression Inventory, BDI; Beck, 1978) and anxiety symptomatology (State-Trait Anxiety Inventory, form Y-2, STAI; Spielberger et al., 1977), and nicotine dependence [Fagerstrom Tolerance Test for Nicotine Dependency (FTND; Fagerstrom et al., 1991)]. The total number of cigarettes currently smoked per day, number of years of smoking at the current level and over lifetime were also recorded, and pack years [i.e., (number of cigarettes per day/20) × lifetime number of years of smoking] calculated for smoking ALC.

### Neuropsychological assessment

Participants completed a comprehensive battery, which evaluated domains of neurocognitive function previously reported to be affected by AUD (Oscar-Berman, 2000; Rourke and Loberg, 2009) and chronic cigarette smoking (Durazzo et al., 2007a; Swan and Lessov-Schlaggar, 2007). Smoking ALC were allowed to smoke *ad libitum* prior to assessment and to take smoking breaks during testing if requested. The neurocognitive domains evaluated and the constituent measures were as follows: *Executive skills*: Short Categories Test (Wetzel and Boll, 1987), color-word portion of the Stroop Test (Golden, 1978), Trail Making Test part B (Reitan and Wolfson, 1985), Wechsler Adult Intelligence Scale 3rd Edition (WAIS-III) Similarities (Wechsler, 1997), Wisconsin Card Sorting Test-64: Computer Version 2-Research Edition (Kongs et al., 2000) non-perseverative errors, perseverative errors, and perseverative responses *General intelligence*: Ward-7 Full Scale IQ (Axelrod et al., 2001; based on WAIS-III Arithmetic, Block Design, Digit Span, Digit Symbol, Information, Picture Completion, and Similarities subtests; Wechsler, 1997). *Learning and memory*: Auditory-verbal: California Verbal Learning Test-II (Delis et al., 2000), Immediate Recall trials 1–5 (learning), Short and Long Delay Free Recall (memory). Visuospatial: Brief Visuospatial Memory Test-Revised (Benedict, 1997), Total Recall (learning), and Delayed Recall (memory). *Processing speed*: WAIS-III Digit Symbol, Stroop Color and Word (Golden, 1978), WAIS-III Symbol Search (Wechsler, 1997), Trail Making Test-A (Reitan and Wolfson, 1985). *Visuospatial skills*: WAIS-III Block Design; Luria–Nebraska Item 99 (Golden et al., 1978). *Working memory*: WAIS-III Arithmetic, WAIS-III Digit Span. *Cognitive efficiency*: this domain consisted of all tests that were timed, or in which the time to complete the task influenced the score achieved, and was calculated by averaging the individual *z*-scores of those measures (see below). Timed tests included the Luria–Nebraska Item 99 ratio, Stroop word, color, and color-word tests, Trails A and B and WAIS-III Arithmetic, Block Design, Digit Symbol, Picture Completion, and Symbol Search. Higher scores on these measures reflect better speed and accuracy on principally non-verbal tasks. The cognitive efficiency domain is an approximation of the concept of cognitive efficiency previously described by Glenn and Parsons (1992) and Nixon et al. (1995, 1998). Premorbid verbal intelligence was estimated with the American National Adult Reading Test (Grober and Sliwinski, 1991). For the Luria–Nebraska Item 99, the number correct (maximum possible = 8) was divided by the time required to complete the task. This ratio was used due to the low ceiling for the number of correct responses (i.e., most participants achieved a score of 6 or better), which resulted in a highly skewed and non-Gaussian distribution. The ratio of number correct to time to complete the Luria 99 was normally distributed.

Raw scores for all neurocognitive measures, except the Luria–Nebraska Item 99 ratio, were converted to age-adjusted standardized scores via the normative data accompanying the particular measure (i.e., BVMT-R, CVLT-II, Short Categories Test, Stroop Color-Word Test, WAIS-III subtests) or age and education [(WCST-64 variables; Trails A and B via Heaton Compendium Norms (Heaton et al., 1991)]. Standardized scores were transformed to *z*-scores for all measures. For the Luria–Nebraska Item 99 ratio, raw scores were converted to *z*-scores based on the performance of 32 non-smoking light drinking controls, as there are no published norms available for this measure. A global neurocognitive functioning score was calculated from the arithmetic mean of *z*-scores for all of the individual domains.

### Genotyping

Genomic DNA was isolated from whole blood. The SNPs were assayed using TaqMan genotyping assays from Applied Biosystems, Foster City, CA, USA. SNP assays were performed using a reaction volume of 15 μl, which consisted of 7.5 μl of TaqMan 2X universal master mix, 0.38 μl of 20X TaqMan pre-designed SNP genotyping assay, 6.14 μl of nuclease-free water, and 1 μl genomic DNA. After PCR amplification as per manufacturer’s recommendations, SNP genotypes were determined by allelic discrimination using the ABI-7500 instrument. BDNF (χ^2^ = 0.79, *p* = 0.37) and COMT (χ^2^ = 0.01, *p* = 0.92) were in Hardy–Weinberg equilibrium (see Table 2).

**Table 2:** **Genotype frequency for BDNF Val66Met, COMT Val158Met**.

SNP	Genotype	Frequency	Percent
BDNF (rs6265)	Val/Val	47	67.1
	Val/Met	22	31.4
	Met/Met	1	1.5
COMT (rs4680)	Val/Val	21	30.0
	Val/Met	35	50.0
	Met/Met	14	20.0

*SNP, single nucleotide polymorphism. All genotypes were in Hardy–Weinberg equilibrium (χ^2^ < 0.83, *p* > 0.36)*.

### Data analyses

Multivariate analyses of covariance (MANCOVA) examined effects of BDNF and COMT genotypes and smoking status on the 11 domains of neurocognition (see Table 3 for list of domains), with age, AMNART, and lifetime average drinks per month as primary covariates. In our previous work with this alcohol dependent cohort, age accounted for a significant amount of the variance in neurocognition despite the use of age-corrected norms (Durazzo et al., 2008, 2010a); therefore, age was also used as a covariate in this study. Significant MANCOVA omnibus effects (*p* = 0.05) for genotypes and smoking status were followed-up with pairwise *t*-tests. To control for the potential influence of medical (primarily hypertension and positivity for the hepatitis C antibody), psychiatric (primarily unipolar mood disorders), and substance abuse history on neurocognition, pairwise comparisons achieving statistical significance were reanalyzed using medical, psychiatric, and substance use comorbidities, individually, as additional covariates. Significance levels of all pairwise comparisons were adjusted for multiplicity of tests. Alpha levels (*p* = 0.05) for pairwise comparisons for BDNF and COMT genotypes and smoking status were adjusted for the number of neurocognitive domains evaluated (i.e., 11) and the average intercorrelation among the domains (i.e., *r* = 0.55), resulting in a corrected *p*-values of 0.017 (see Sankoh et al., 1997). Effect sizes (ES) for pairwise comparisons were calculated via Cohen’s *d* (Cohen, 1988). For smoking ALC, associations (i.e., semi-partial correlations) between the 11 neurocognitive domains, genotypes, lifetime average drinks per month, and lifetime years of smoking were examined with multiple linear regression (all predictors simultaneously entered into the model). Analyses were completed with SPSS v18.0.

**Table 3:** **Associations between neurocognitive domains (age-corrected) and lifetime years of smoking for smoking ALC (*n* = 39)**.

Neurocognitive domain	Lifetime years of smoking
Auditory-verbal learning	−0.39**
Auditory-verbal memory	−0.38*
Cognitive efficiency	−0.37**
Executive skills	−0.23
General intelligence	−0.27*
Processing speed	−0.30*
Visuospatial learning	−0.50**
Visuospatial memory	−0.45**
Visuospatial skills	−0.43**
Working memory	−0.16
Global neurocognition	−0.49**

*p < 0.05; **p < 0.01; all tests two-tailed. Correlations are semi-partial coefficients controlling for AMNART, lifetime average drinks per month, BDNF, and COMT genotypes.

## Results

### Participant characterization

Participants were 51.0 ± 10.0 years of age, had 14.0 ± 2.2 years of formal education and were abstinent for 33 ± 9 days at the time of study. Eighty percent of ALC participants were Caucasian, 13% African American, 4% Latino, 2% Native American, and 1% Pacific Islander. See Table 1 for additional demographics and clinical measures.

### Smoking status, COMT and BDNF genotypes, and neurocognitive function

Multivariate analyses of covariance indicated significant omnibus effects for smoking status [*F* (10, 53) = 3.18, *p* < 0.003], COMT genotype [*F* (20, 108) = 1.77, *p* = 0.042], age [*F* (10, 53) = 2.97, *p* = 0.005], and AMNART [*F* (10, 53) = 11.74, *p* < 0.001]. BDNF genotype and lifetime average drinks per month were not significant predictors of neurocognition. Inspection of pairwise tests across domains for BDNF Val homozygotes versus heterozygotes revealed all comparisons were *p* > 0.15, with trivial ES (all <0.16).

Pairwise comparisons indicated smoking ALC performed worse than non-smoking ALC on the following domains of functioning: auditory-verbal learning (*p* < 0.001; ES = 0.83), auditory-verbal memory (*p* < 0.001; ES = 0.87), cognitive efficiency (*p* < 0.001; ES = 0.97), general intelligence (*p* < 0.001; ES = 0.92), processing speed (*p* < 0.001; ES = 0.97), visuospatial learning (*p* = 0.001; ES = 0.75), visuospatial memory (*p* = 0.007; ES = 0.60), and global neurocognition (*p* < 0.001; ES = 1.09). Smoking ALC showed a trend for lower executive skills (*p* = 0.05; ES = 0.40). Controlling the above listed pairwise tests for COMT, medical, psychiatric, and substance abuse comorbidities did not appreciably alter the above *p*-values or ES for differences between smoking and non-smoking ALC.

Pairwise comparisons showed COMT Met homozygotes (i.e., Met/Met) were superior to Val homozygotes (i.e., Val/Val) on executive skills (*p* = 0.013, ES = 0.75) and showed trends for higher general intelligence (*p* = 0.035, ES = 0.61) and visuospatial skills (*p* = 0.022, ES = 0.69) than Val homozygotes. Val/Met heterozygotes demonstrated a significantly better performance on the general intelligence domain than Val homozygotes (*p* = 0.014, ES = 0.45). Val homozygotes performed significantly better than Val/Met on auditory-verbal memory (*p* = 0.012, ES = 0.65). Controlling the above listed pairwise tests for smoking status, medical, psychiatric, and substance abuse comorbidities did not alter the above reported results.

### Associations of genotypes with alcohol consumption and lifetime years of smoking

No significant associations were observed among BDNF and COMT genotypes, alcohol consumption measures, and the 11 neurocognitive domains. For smoking ALC, higher lifetime years of smoking showed moderate to strong inverse relationships with performance on multiple neurocognitive domains after controlling for AMNART, lifetime average drinks per month, BDNF and COMT genotypes (see Table 3). There were no relationships between FTND score (i.e., level of nicotine dependence) and any neurocognitive domain.

## Discussion

The primary findings from this cohort of primarily male, treatment-seeking alcohol dependent individuals with approximately 1 month of abstinence from alcohol were as follows: (1) smoking ALC demonstrated significantly poorer performance than non-smoking ALC on multiple domains of neurocognition *after* controlling for COMT and BDNF genotypes and medical, psychiatric, and substance abuse comorbidities; (2) in smoking ALC, greater number of lifetime years of smoking was associated with worse performance on multiple neurocognitive domains; (3) COMT genotype was significantly associated with measures of executive skills, general intelligence, and visuospatial memory; and (4) the BDNF Val66Met polymorphism was not a significant predictor of any neurocognitive domain.

Chronic cigarette smoking in this cohort of alcohol dependent individuals in early recovery was a robust predictor of performance in multiple domains of neurocognition after controlling for BDNF and COMT genotypes, lifetime alcohol consumption, age, and AMNART. The pattern of inferior performance of smoking ALC relative to non-smoking ALC and the moderate to strong ES for the group differences are consistent with our previous research (Durazzo et al., 2008, 2010a) as well as with findings from other studies (e.g., Glass et al., 2006, 2009). Additionally, in smoking ALC, the relationships of greater number of years of lifetime smoking to age-adjusted scores on multiple neurocognitive domains remained significant and robust after controlling for BDNF and COMT, lifetime alcohol consumption, and comorbid conditions. Taken together, this suggests that the inferior performance of smoking compared to non-smoking ALC and the moderate to strong associations of lifetime years of smoking with neurocognition in smoking ALC were not mediated by the SNPs investigated, cumulative amount of alcohol consumed over lifetime, or conditions that are highly comorbid with AUD.

When assessing the effects of chronic cigarette smoking on neurocognition, it is important to distinguish between the effects of acute ingestion, metabolism and withdrawal of nicotine, and the influence of chronic exposure to the multitude of noxious compounds contained in cigarette smoke. Acute nicotine administration has been found to transiently improve some areas of neurocognition in healthy non-smokers and individuals with attention deficit hyperactivity disorder and schizophrenia-spectrum disorders, predominantly on measures of sustained attention and working memory (Rezvani and Levin, 2001; Sacco et al., 2004; Mansvelder et al., 2006). Acute nicotine administration in nicotine deprived smokers is associated with improved cognitive task performance (Mendrek et al., 2006; Parrott, 2006), whereas several studies report decrements in neurocognitive performance with nicotine administration to non-smokers (see Mansvelder et al., 2006 for review). A recent meta-analysis conducted by Heishman et al. (2010) suggests that acute smoking or nicotine consumption, independent of withdrawal effects, are associated with enhanced performance in the following domains of function: fine motor skills, alerting attention accuracy and response time, orienting attention reaction time, short-term episodic memory accuracy, and working memory reaction time (but not accuracy). There is limited placebo controlled research assessing the effects of acute nicotine administration in AUD. In alcohol dependent smokers with 40 ± 17 days of abstinence, a high acute nicotine dose administered via transdermal patch (14 and 21 mg for females and males, respectively), was related to greater accuracy on a measure of vigilance and working memory than a low nicotine dose (7 mg; Boissoneault et al., 2011), but neither the high nor the low nicotine dose influenced immediate or delayed auditory-verbal memory performance (Gilbertson et al., 2011). Greater pack years (a composite measure of smoking intensity and chronicity), was related to longer reaction times and lower accuracy on the vigilance and working memory task (Boissoneault et al., 2011). Similarly, in community-based samples of men with a lifetime history of alcohol dependence, higher pack years were inversely related to measures of cognitive proficiency and general intelligence (Glass et al., 2006) and both smoking and alcoholism severities were inversely related to executive function (Glass et al., 2009). In this study, longer lifetime smoking duration was associated with poorer performance on multiple neurocognitive domains, which is consistent with the findings for pack years in the above studies. sALC in this study were allowed to smoke *ad libitum* prior to assessment and to take smoke breaks during the assessment. The plasma half-life of nicotine is about 2 h (Nakajima and Yokoi, 2005), and, with a 2 h half-life, plasma nicotine levels will accrue (e.g., 3 or more half-lives) with regular smoking during waking hours (Hukkanen et al., 2005); therefore, nicotine withdrawal likely did not confound any of our findings (for review see Sacco et al., 2004). Taken together, acute nicotine administration in smoking AUD may facilitate performance on some aspects of neurocognition; however, it appears that increasing smoking intensity and/or chronicity in AUD is robustly related to poorer performance on multiple neurocognitive functions and may mitigate any enhancing effects of acute nicotine consumption, particularly with greater levels of smoking severity and/or chronicity. For further discussion of potential mechanisms associated with the neurocognitive and neurobiological effects of chronic cigarette smoking in AUD and non-clinical samples (see Durazzo and Meyerhoff, 2007; Durazzo et al., 2010b).

The most consistent finding for COMT in this alcohol dependent cohort was that Met allele carriers performed better than Val homozygotes on measures of executive skills and general intelligence. Specifically, Met homozygotes and Val/Met heterozygotes performed significantly better than Val homozygotes on the executive skills and general intelligence domains, respectively. Met homozygotes showed trends for better performance than Val homozygotes on the general intelligence and visuospatial skills domains. Moderate ES were apparent for the differences between COMT Met carriers and Val homozygotes. There were no significant differences between COMT Met homozygotes and Val/Met heterozygotes, and Val homozygotes were not superior to Met homozygotes on any neurocognitive domain. Our COMT findings for the executive skills domain in this alcohol dependent cohort are consistent with studies of the COMT rs4680 SNP in normal controls and individuals with neuropsychiatric disorders, which reported that Met homozygotes were superior to Val homozygotes on measures of executive skills (Savitz et al., 2006; Wishart et al., 2011). With respect to specific measures of executive skills, studies have found Met homozygotes made significantly less perseverative responses or perseverative errors on the WCST than Val homozygotes across cohorts of normal controls, individuals at risk for schizophrenia, and schizophrenics (Joober et al., 2002; Malhotra et al., 2002; Mattay et al., 2003; Rosa et al., 2004). COMT Met homozygotes in this report also made less perseverative errors and perseverative responses on the WCST than Val homozygotes (*p* < 0.05), after controlling for BDNF genotype, smoking status, lifetime alcohol consumption, age, and AMNART (data for individual tests not shown). The influence of the COMT Val158Met SNP on neurocognition may be related to its effects on the regulation of tonic and phasic dopamine activity (DA) in the frontal lobe neocortex. The G → A missense mutation in this SNP translates into a substitution of Val by Met at codon 158. Physiologically, the Val158Met SNP affects the thermostability of the COMT enzyme in a Met dose-dependent fashion such that Met homozygotes demonstrate approximately 50% reduction in enzymatic activity in the frontal lobe cortex (see Dickinson and Elvevag, 2009). The decreased enzymatic activity of COMT Met allele carriers is thought to result in higher tonic and more stable DA concentrations at paralimbic and neocortical D1 receptors and lower phasic alterations in subcortical DA levels, which is suggested to relate to better and more consistent performance on abilities subserved by the anterior frontal-subcortical circuits, particularly executive skills and working memory (see Bilder et al., 2004; Dickinson and Elvevag, 2009). The superior performance of Met carriers relative to Val homozygotes on measures of executive and intellectual skills is consistent with the suggested effects of COMT genotype on tonic-phasic DA neurotransmission in anterior frontal-subcortical circuits subserving higher order neurocognitive functions. Contrary to previous studies, the BDNF Val66Met polymorphism was not a significant predictor of any neurocognitive domain. ES for pairwise comparisons of BDNF genotypes across the 11 domains were trivial (0.01–0.15), which suggests the lack of significant findings in Val homozygotes and Met Carriers were not a function of insufficient statistical power.

Age was a significant predictor of all domains except of auditory-verbal learning and memory and working memory, *despite the use of age-adjusted norms*. Fast, flexible, and accurate responses are required for better scores on the predominantly non-verbal/visuospatial tasks comprising the cognitive efficiency, processing speed, and visuospatial skills domains, as well as on WAIS-III non-verbal tasks contributing to the general intelligence domain. Research on normal age-related changes in neurocognition suggests decreasing information processing speed is significantly related to the declines in learning, memory, and visuospatial abilities with increasing age (Salthouse, 1996, 2000; Christensen, 2001; Finkel et al., 2007; Kochunov et al., 2010). Overall, the age effects observed in this study are congruent with the “premature aging” hypothesis in AUD (Oscar-Berman, 2000). It is also noteworthy that, in this report, and in our earlier work (Durazzo et al., 2007c, 2008, 2010a) measures of alcohol consumption were not associated with neurocognition. This is consistent with other research that found measures of alcohol consumption quantity/frequency were weakly or not related to neurocognition (Schafer et al., 1991; Beatty et al., 1995, 2000; Eckardt et al., 1998; Horner et al., 1999; Sullivan et al., 2000).

This study has limitations that may influence the generalizability of the findings. The sample size of this study was modest, which did not permit a full factorial examination of all predictors (e.g., gene × gene interactions) and possibly led to inadequate power to detect other potential relationships between COMT and the neurocognitive domains investigated. We did not assess for personality disorders, which may contribute to the neurocognitive and neurobiological abnormalities observed in AUD (Eckardt et al., 1995; Kuruoglu et al., 1996; Giancola and Moss, 1998; Costa et al., 2000). Results may have also been influenced by factors not directly assessed in this study, such as diet, exercise, and exposure to environmental cigarette smoke or other premorbid/genetic variables. Finally, the majority of participants were males recruited from the San Francisco VA Medical Center, which did not allow for the examination of the potential effects of sex on neurocognition.

In summary, chronic cigarette smoking was strongly related to poorer performance on multiple neurocognitive domains, while the COMT Val158Met polymorphism showed significant associations with three domains (executive skills, general intelligence, and auditory-verbal memory) in this cohort of short-term abstinent alcohol dependent individuals. Importantly, our results indicate that the inferior performance demonstrated by smoking compared to non-smoking ALC was not mediated by the SNPs investigated, alcohol consumption, or comorbid medical and psychiatric conditions. The current findings reinforce our previous work that indicates consideration of smoking status and other prevalent comorbid conditions in AUD is critical to fully appreciate how this clinical syndrome influences neurocognition. Our results for the relationships of COMT polymorphism to neurocognition in AUD were consistent with findings in normal controls and individuals with schizophrenia-spectrum disorders. Research investigating the influence of BDNF and COMT on neurocognitive recovery during sustained abstinence from alcohol in this cohort is clearly indicated. Cigarette smoking is a modifiable health risk that is directly associated with at least 440,000 deaths in the United States alone and 10 million annual deaths worldwide, with greater mortality and morbidity among those with substance use disorders, mood disorders, and schizophrenia (see Durazzo and Meyerhoff, 2007 for review). This study provides clinicians with additional information on the adverse consequences of chronic smoking in those seeking treatment for AUD. In the face of high mortality from cigarette smoking in AUD (Hurt et al., 1996), the data from this report in conjunction with other neurocognitive and neuroimaging studies (see Durazzo and Meyerhoff, 2007; Durazzo et al., 2010b), lend strong support to the expanding clinical movement (which is standard practice at the San Francisco VA Medical Center) to make smoking cessation programs available to smokers at the inception of treatment for alcohol/substance use disorders.

## Conflict of Interest Statement

The authors declare that the research was conducted in the absence of any commercial or financial relationships that could be construed as a potential conflict of interest.
